# ECAT1 is essential for human oocyte maturation and pre-implantation development of the resulting embryos

**DOI:** 10.1038/srep38192

**Published:** 2016-12-05

**Authors:** Changyu Liu, Min Li, Tianjie Li, Hongcui Zhao, Jin Huang, Yun Wang, Qian Gao, Yang Yu, Qinghua Shi

**Affiliations:** 1 Molecular and Cell Genetics Laboratory; The CAS Key Laboratory of Innate Immunity and Chronic Disease; Hefei National Laboratory for Physical Sciences at Microscale; School of Life Sciences, University of Science and Technology of China, Hefei 230027, China; 2 Beijing Key Laboratory of Reproductive Endocrinology and Assisted Reproductive Technology and Key Laboratory of Assisted Reproduction, Ministry of Education, Center of Reproductive Medicine, Department of Obstetrics and Gynecology, Peking University Third Hospital, Beijing 100191, China

## Abstract

ECAT1 is a subunit of the subcortical maternal complex that is required for cell cycle progression during pre-implantation embryonic development; however, its exact function remains to be elucidated. Here we investigated the expression of ECAT1 in human ovarian tissue, oocytes and pre-implantation embryos and assessed its function by using RNA interference (RNAi) in oocytes. *ECAT1* mRNA was highly expressed in human oocytes and zygotes, as well as in two-cell, four-cell and eight-cell embryos, but declined significantly in morulae and blastocysts. ECAT1 was expressed in the cytoplasm of oocytes and pre-implantation embryos and was localized more specifically in the cortical region than in the inner cytoplasm. RNAi experiments demonstrated that down-regulation of ECAT1 expression not only impaired spindle assembly and reduced maturation and fertilization rates of human oocytes but also decreased the cleavage rate of the resulting zygotes. In conclusion, our study indicates that ECAT1 may play a role in meiotic progression by maintaining the accuracy of spindle assembly in human oocytes, thus promoting oocyte maturation and subsequent development of the embryo.

Oocyte maturation is a prolonged and complex process, during which oocytes store an abundance of maternal RNAs and proteins that are essential for successful fertilization and pre-implantation development^1^,^2^. Oocytes become arrested at the diplotene stage of meiotic prophase after their formation in the fetal gonad and then resume meiosis and progress to the metaphase stage of the second meiotic division (MII)^3^,^4^,^5^. The process of oocyte maturation includes nuclear maturation, cytoplasmic maturation and epigenetic maturation^6^,^7^. Appropriate cross-talk between the nucleus and cytoplasm determines the maturation of the oocyte and the developmental potential of the resulting embryo.

Oocyte maturation requires precise regulation and a series of interactions by maternal genes and proteins that play key roles not only in the process of maturation but also in embryonic development before complete zygotic genome activation^8^. In previous studies, although some maternal genes with roles in oocyte maturation and embryonic development have been characterized, most of those genes were implicated mainly from mouse data^9^,^10^. With the emergence of single-cell sequencing technology, the transcriptome and methylome of human oocytes and pre-implantation embryos have been determined, and many human genes that are differentially expressed during early development have been isolated^11^,^12^,^13^,^14^. Therefore, we can now examine genes directly from the relevant human database and investigate their functions.

Recently, a subcortical maternal complex (SCMC), which includes FILIA, FLOPED, MATER and TLE6, has been identified. It is assembled during oocyte growth and is essential for zygotes to progress beyond the first embryonic cell division in mice^15^,^16^,^17^,^18^,^19^. *Filia*, a single-copy gene with two mRNA isoforms (1.6 and 1.2 kb), is located on mouse chromosome 9qD and encodes the binding partner of MATER; its transcripts are specifically detected in ovaries but not in testes or other somatic tissues^15^,^17^. The absence of FILIA reduces fecundity, impairs pre-implantation development of the embryo with a high incidence of aneuploidy and contributes to low-quality cell cycle progression^16^.

ECAT1, the human homolog of mouse Filia, is localized on human chromosome 6q13. ECAT1 is a member of the KH protein family, which also includes KHDC1, DPPA5 and OOEP, and is characterized by the presence of an N-terminal K homology domain; its members may have functions that involve binding RNA^20^,^21^. The genes of the KH protein family are specifically expressed in oocytes and/or embryonic stem cells and have undergone rapid evolution, as they are not present in fish, chicken or opossum^20^,^22^,^23^. ECAT1 mRNA is highly expressed in human oocytes at the germinal vesicle (GV) stage, is expressed at lower levels in oocytes through the metaphase II stage and is almost absent in pre-implantation embryos; therefore ECAT1 is predicted to be a maternal gene^24^. ECAT1 has also been implicated in human diseases, such as hydatidiform mole which is a specific form of pregnancy loss and is characterized by an absence of or abnormal embryonic development, hydropic degeneration of chorionic villi and excessive proliferation of trophoblast cells^25^. Mutations in human ECAT1 are associated with familial biparental hydatidiform mole and recurrent hydatidiform moles, and thus ECAT1 may function as a regulator of the cell cycle^24^,^25^. Regarding the rapid cell cycle progression during mammalian pre-implantation embryonic development, the mechanism by which ECAT1 affects human oocyte maturation, fertilization and embryonic development remains unknown.

In the present study, we investigated the mRNA expression profile of *ECAT1* in human tissue, oocytes and pre-implantation embryos. We localized ECAT1 in fetal and adult ovaries by immunohistochemistry as well as in oocytes and pre-implantation embryos by immunofluorescence. Function of ECAT1 was confirmed by down-regulating ECAT1 expression via short interfering RNA (siRNA) injection into human immature oocytes to study the role of ECAT1 in oocyte maturation, fertilization and pre-implantation embryonic development. The effects of ECAT1 knockdown were analyzed using oocyte maturation and the efficiency of embryonic development and spindle assembly.

## Results

### ECAT1 mRNA is highly expressed in human ovaries

**Figure 1:**
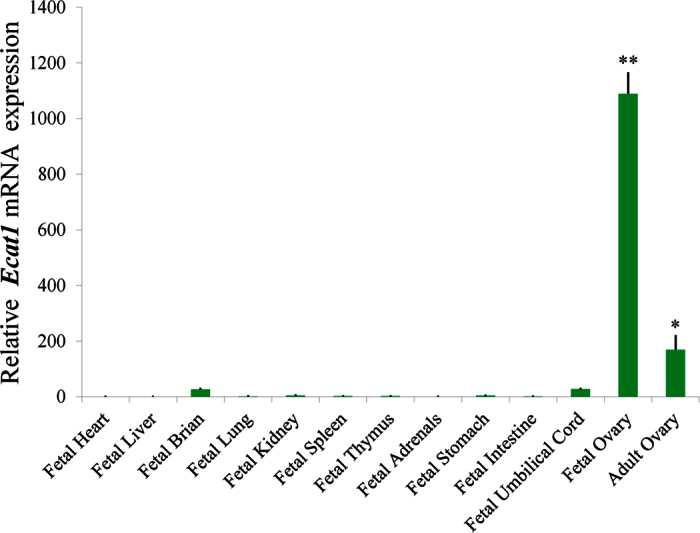
Relative mRNA expression of *ECAT1* in human tissues. qPCR was performed with total RNA extracted from 13 human tissues including fetal heart, fetal liver, fetal brain, fetal lung, fetal kidney, fetal spleen, fetal thymus, fetal adrenal, fetal stomach, fetal intestine, fetal umbilical cord, fetal ovary and adult ovary. All bar graphs show the mean ± s.e.m. *P < 0.05, **P < 0.001.

Tissues from three aborted female fetuses were collected at gestational ages of 19–22 weeks after legal, voluntary termination of pregnancy due to maternal physical disorders at Peking University Third Hospital. Human adult ovaries from a single individual were also collected with informed consent at Peking University Third Hospital. *ECAT1* mRNA was detected in 13 tissues (fetal heart, fetal liver, fetal brain, fetal lung, fetal kidney, fetal spleen, fetal thymus, fetal adrenal, fetal stomach, fetal intestine, fetal umbilical cord, fetal ovary and adult ovary) by quantitative real-time PCR (qPCR). *ECAT1* was highly expressed in human fetal ovary, at levels six-fold higher than its expression in adult ovary (Fig. 1). Low-level expression was also detected in the fetal brain and umbilical cord; expression was barely detected in the other nine tissues (Fig. 1).

### ECAT1 is expressed in ovaries and GV oocytes

**Figure 2:**
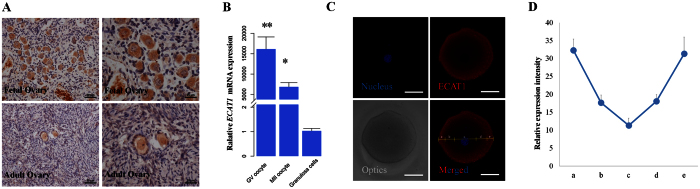
Intraovarian and intraoocyte localization of ECAT1. (**A**) ECAT1 was expressed in human fetal and adult ovaries. (**B**) *ECAT1* mRNA was extensively expressed in oocytes at germinal vesicle and metaphase II stages as compared with granulosa cells. (**C**) Localization of ECAT1 in human oocytes by immunofluorescence. As shown in the image in the bottom right corner, a and e are defined as cortical cytoplasm, whereas b, c and d are defined as the inner cytoplasm region (a/b/c/d/e = 1:1.5:3:1.5:1). Bar is 50 μm. (**D**) Intensity of ECAT1 staining was analyzed in the five regions defined in (**C**). All bar graphs show the mean ± s.e.m.

To investigate the localization of ECAT1 in human ovary, we carried out immunohistochemical staining of fetal and adult ovarian tissue. ECAT1 was notably detected in the oocyte cytoplasm of fetal and adult ovaries (Fig. 2A). Immunoreactivity was less obvious in granulosa cells of fetal and adult ovaries (Fig. 2A), which is consistent with mRNA expression of *ECAT1* in granulosa cells and oocytes at GV and Metaphase II (MII) stages (Fig. 2B). Immunocytochemical analysis of ECAT1 protein in germinal vesicle (GV) oocytes using confocal fluorescence microscopy demonstrated that ECAT1 protein was present throughout the cytoplasm of oocytes, with higher expression in the subcortex (Fig. 2C and D).

### ECAT1 is expressed in human oocytes and pre-implantation embryos

**Figure 3:**
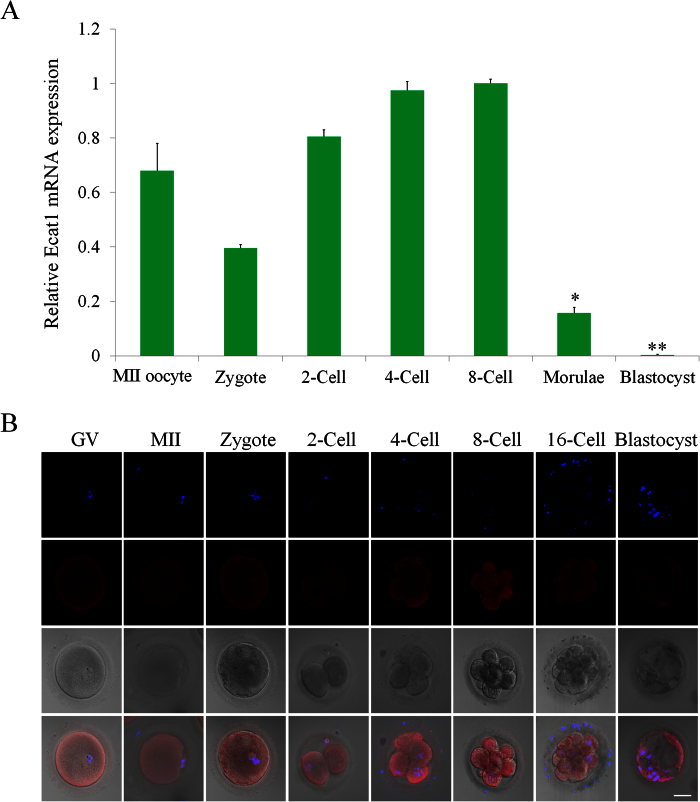
mRNA expression and protein localization of ECAT1 in human oocytes and pre-implantation embryos. (**A**) Relative *ECAT1* mRNA expression was higher in oocytes through eight-cell embryos but declined after the morula stage. All bar graphs show the mean ± s.e.m. *P < 0.05, **P < 0.001. (**B**) ECAT1 was present in the cytoplasm of oocytes and pre-implantation embryos and was continuously expressed from oocytes to the blastocyst stage. Bar is 50 μm.

To determine the function of ECAT1 during the early development of human embryos, the temporal expression pattern of *ECAT1* in human MII oocytes and pre-implantation embryos was first investigated by single-cell qPCR. *ECAT1* mRNA was abundant in the oocyte through the eight-cell embryo. The amount of *ECAT1* mRNA declined from oocyte to zygote and then began to increase until the eight-cell embryo. However, the expression of *ECAT1* mRNA declined in embryos collected at the morula stage, and mRNA was barely detectable at the blastocyst stage (Fig. 3A).

To investigate the sublocalization of ECAT1, its temporal and spatial expression was examined by immunofluorescence in human oocytes and pre-implantation embryos. Immunocytochemical analysis demonstrated that ECAT1 was abundant in GV- and MII-stage oocytes as well as in embryos from the zygote to blastocyst stage (Fig. 3B). ECAT1 was expressed across the cytoplasm of pre-implantation embryos, with higher levels in the subcortex (Fig. 3B), which is similar to its localization in oocytes. Therefore, ECAT1 may play a role in oocyte maturation and pre-implantation embryonic development.

### Down-regulation of ECAT1 reduces the rate of oocyte maturation *in vitro*

**Figure 4:**
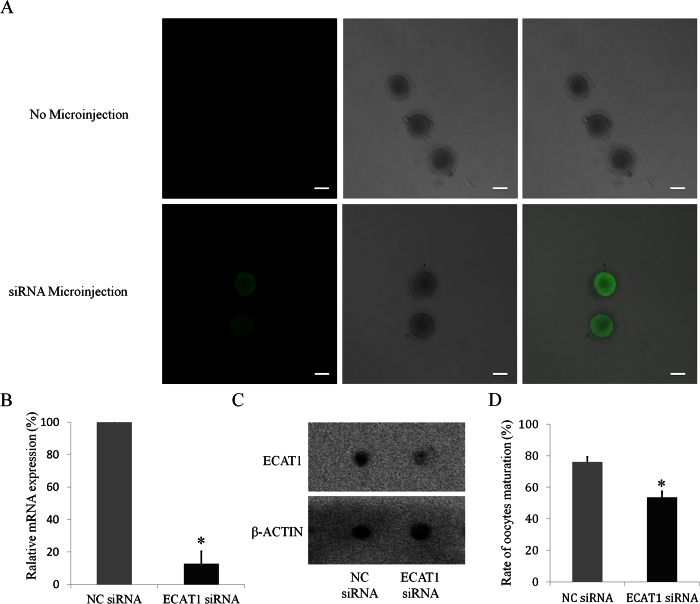
Effect of ECAT1 siRNA microinjection on ECAT1 expression and oocyte maturation. (**A**) The successfully injected oocytes are shown based on their green fluorescence. Bar is 100 μm. (**B**) *ECAT1* mRNA expression was significantly lower in the oocytes with ECAT1 siRNA injection. (**C**) ECAT1 expression was also lower in the oocytes with ECAT1 siRNA injection. (**D**) The oocyte maturation rate was significantly decreased in the oocytes with ECAT1 siRNA injection as compared with controls. All bar graphs show the mean ± s.e.m. *P < 0.05.

We then investigated the role of ECAT1 during maturation, fertilization and development by RNAi experiments. Three ECAT1 siRNA species targeting different regions of the ECAT1 transcript were produced *in vitro*, and we mixed the three siRNA species and microinjected them into GV oocytes. After siRNA injection, the oocytes were cultured for 24 h. Successful siRNA injection into human GV oocytes was identified by green fluorescence (labeled with 6-carboxyfluorescein) using confocal fluorescence microscopy (Fig. 4A). Compared with negative control (NC) siRNA–injected oocytes, microinjection of the cocktail of ECAT1 siRNAs after 42 h significantly reduced *ECAT1* mRNA in oocytes by ~90% (Fig. 4B). Furthermore, ECAT1 was also significantly reduced in the ECAT1 siRNA group based on dot-blot analysis of eight oocytes from each group at 48 h post-injection (Fig. 4C).

Oocyte maturation is morphologically marked by extrusion of the first polar body and occurrence of germinal vesicle breakdown (GVBD)^26^. A total of 180 oocytes were microinjected with ECAT1 siRNA, of which 154 survived, and 83 of the surviving oocytes matured *in vitro* Table 1). In the NC siRNA group, 60 of 71 microinjected oocytes survived, and 47 matured *in vitro*. The maturation rate of human GV oocytes in the ECAT1 siRNA group (54%) was significantly lower than that in the NC siRNA group (76%; Fig. 4D, P < 0.05).

### ECAT1 knockdown reduces the rate of fertilization and cleavage

**Figure 5:**
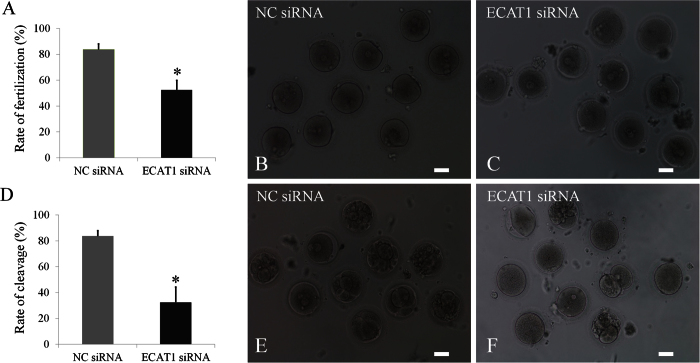
Knockdown of ECAT1 expression significantly impaired embryo fertilization and cleavage efficiency. (**A**) Significantly fewer oocytes injected with ECAT1 siRNA were fertilized relative to the NC siRNA oocytes. (**B,C**) Representative images for fertilization are shown for oocytes with NC siRNA injection (**B**) and oocytes with ECAT1 siRNA injection (**C**). (**D**) A significantly lower cleavage rate occurred in the embryos from oocytes injected with ECAT1 siRNA as compared with those from the NC siRNA oocytes. (**E,F**) Representative images for cleavage are shown for day 3 embryos from oocytes injected with NC siRNA (**E**) and day 3 embryos from oocytes injected with ECAT1 siRNA (**F**). Bar is 100 μm.

To determine whether knockdown of ECAT1 in human oocytes has any effect on the fertilization frequency, we performed intracytoplasmic sperm injection (ICSI) using a subset of those oocytes that had successfully matured *in vitro* by 24 h after siRNA microinjection. In the NC siRNA group, 84% (26/31) of the oocytes were successfully fertilized as indicated by the formation of two pronuclei within 12 h, whereas 54% (14/26) of the oocytes were fertilized in the ECAT1 siRNA group (Table 2). The fertilization of human MII oocytes in the ECAT1 siRNA group was significantly lower than that in the NC siRNA group (Fig. 5A, P < 0.01, and Fig. 5B and C).

Next, we examined whether the knockdown of ECAT1 in human GV oocytes has any effect on the cleavage rate within 24 h after ICSI. In the NC siRNA group, 84% (26/31) of the oocytes reached the two-cell stage, whereas only 31% (8/26) of the oocytes reached the two-cell stage in the ECAT1 siRNA group (Table 2). The cleavage rate of the ECAT1 siRNA group was significantly lower than that in the NC siRNA group (Fig. 5D, P < 0.01, and Fig. 5E and F). Thus, knockdown of ECAT1 in human GV oocytes impaired their subsequent fertilization and cleavage.

### ECAT1 knockdown increases the rate of abnormal spindles in MII oocytes

**Figure 6:**
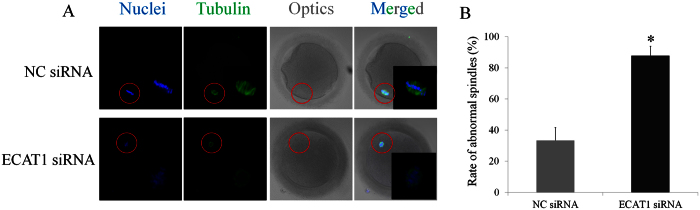
Correlation between spindle assembly and ECAT1 expression. (**A**) Representative images for spindle assembly in the oocytes with or without ECAT1 siRNA injection. (**B**) A significantly increased number of abnormal spindles was found in the oocytes with ECAT1 siRNA injection. All bar graphs show the mean ± s.e.m. *P < 0.05.

To detect the potential mechanism through which ECAT1 knockdown impairs human oocyte maturation and development, we identified the spindle assembly in human oocytes. Oocyte maturation requires the timely resumption of meiosis. During this process, we found that 88% of MII oocytes (n = 17) from the ECAT1 siRNA group had improper spindles, as compared with 33% (n = 14) in the control group (Fig. 6A and B). The spindle assembly checkpoint (SAC) governs proper chromosome segregation during meiosis and mitosis, and spindle abnormalities are often accompanied by chromosome misalignment. We observed a high incidence of chromosome misalignment in the ECAT1 siRNA group (Fig. 6A), indicating that knockdown of ECAT1 may impair SAC function. Therefore, we suggest that the abnormality of spindle formation in MII oocytes resulting from knockdown of ECAT1 may contribute to abnormal fertilization and cleavage.

## Discussion

In the present study, we systematically investigated the expression characteristics and function of ECAT1 in human oocytes. We showed that ECAT1 plays a key role in human oocyte maturation and that down-regulation of ECAT1 expression results in oocytes that fail to mature and develop. These effects are most likely attributed to spindle and chromosome assembly errors.

Our finding that ECAT1 mRNA is highly expressed in human fetal and adult ovaries but is expressed at low levels in other human tissues is consistent with results from previous studies on human and mouse^17^,^27^. It was interesting to note that ECAT1 was localized not only in the cortical cytoplasm but more specifically in the inner cytoplasm. Previous studies using immunofluorescence staining have demonstrated that FILIA, the mouse homolog of human ECAT1, and the other three components (MATER, FLOPED and TLE6) of the SCMC are expressed specifically in the subcortex of mouse oocytes and pre-implantation embryos^15^,^16^,^17^,^28^,^29^. A similar result was subsequently shown in human oocytes and zygotes^27^. In oocytes and pre-implantation embryos of mice and cows, however, MATER, FLOPED and TLE6 are detected throughout the cytoplasm, and the staining signals are enhanced only in the subcortex^18^,^30^,^31^,^32^,^33^,^34^, which is consistent with the ECAT1 staining pattern we noted here. Moreover, Akoury *et al*. suggested that ECAT1 strongly co-localizes with the oocyte cytoskeleton but is confined to the outer cortical region from two-cell to morula-stage embryos, and ECAT1 remains localized to the nucleus and is expressed in both the inner cell mass and the trophoblast layer at the blastocyst stage^35^. As those authors noted, their results were based on stored human oocytes and embryos, whereas our samples were fresh, which may result in the differences in the results of ECAT1 expression between our two studies.

The subcellular localization of cytoplasmic lattice-associated proteins (such as MATER and PADI6) is dependent on the fixation and processing protocols used^32^,^34^. For example, when oocytes are fixed and then incubated with primary antibodies at room temperature for 1 h, MATER localization in GV oocytes is mainly cytoplasmic, whereas its localization is mainly limited to the cortex when oocytes are fixed and incubated in primary antibody at 4 °C overnight.

The analysis of components of the SCMC in embedded ovarian tissue from mouse, cow and human by immunofluorescence or immunohistochemical staining revealed that these factors are primarily localized throughout the cytoplasm^15^,^24^,^27^,^28^,^29^,^30^,^31^,^33^,^36^, which is consistent with our staining pattern for ECAT1 in human ovaries. In addition, the localization of MATER and PADI6 in cross-sections of fixed ovaries and oviducts is primarily limited to the cytoplasm of GV oocytes and two-cell embryos, with no statistical differences between cortical and cytoplasmic signals^32^.

RNAi is a powerful technology that can rapidly suppress specific genes through double-stranded short interfering RNAs (siRNAs) to learn the function of these genes^37^,^38^,^39^,^40^,^41^. Although RNAi has been used in oocytes and pre-implantation embryos of mice, rats, cows and even monkeys^42^,^43^,^44^,^45^, the use of RNAi with human oocytes and pre-implantation embryos has been limited^46^. We mixed three siRNA species targeting *ECAT1* and microinjected them into GV oocytes collected from the couples who were undergoing ICSI cycles. ECAT1 mRNA and protein were significantly reduced after siRNA microinjection, as detected by single-cell qPCR and dot blot methods, respectively, strongly indicating that we could use the RNAi strategy to investigate the role of ECAT1 in oocytes and pre-implantation embryonic development.

*Filia*, the mouse homolog of human *ECAT1*, is one of the maternal effect genes whose absence impairs pre-implantation embryonic development, but not oogenesis, folliculogenesis, meiotic maturation or ovulation^16^. Evidence does, however, suggest that Filia is associated with oocyte maturation. Phosphatidylinositol 3,4,5-trisphosphate [PtdIns(3,4,5)P3] is constitutively synthesized and is required for F-actin organization and spindle translocation during meiosis in mouse oocytes, but the spatial and temporal dynamics of PtdIns(3,4,5)P3 are impaired by depletion of FILIA and MATER^47^. Furthermore, another member of the SCMC, TLE6, is a substrate of protein kinase A (PKA), which increases from the time of germinal vesicle breakdown (GVBD) until the metaphase II arrest, and the blocking of PKA activity results in altered GVBD kinetics, defects in spindle and chromatin dynamics and a decline in the ability of oocytes to reach the MII stage^28^. The other SCMC members (FILIA, MATER and OOEP) may also be substrates of PKA. In our study, the oocyte maturation rates between the ECAT1 knockdown and control groups varied, but within a narrow range, which may be due to the abundance of proteins in oocytes and the fact that they could not be degraded efficiently within a short time.

Disruption of the meiotic spindle results in rearrangement or scattering of chromosomes and may contribute to abnormal fertilization and to aneuploidy after fertilization. Aneuploidy is one of the most commonly observed patterns of abnormal fertilization in humans and is the major reason for pre-implantation embryonic death and spontaneous abortion. The absence of FILIA impairs pre-implantation embryonic development with a high incidence of aneuploidy and leads to low-quality cell cycle progression because of abnormal spindle assemble, which is similar to our observations of pre-implantation embryonic development after knockdown of ECAT1. We also found that knocking down ECAT1 results in a lower frequency of fertilization and first cleavage. Therefore, we suggest that the abnormality of oocyte maturation resulting from knockdown of ECAT1 leads to abnormal spindle formation in MII oocytes and may be part of the reason for abnormal fertilization and cleavage.

In summary, we have detected the expression and localization of ECAT1 in human tissues, which are somewhat different than the expression and localization of FILIA in mice. As ECAT1 is essential for oocyte maturation and fertilization and embryonic cleavage, our study has provided a new molecular target that may facilitate the maturation and development of human oocytes and embryos in assisted reproductive technologies.

## Methods

### Participants and reagents

The present study was approved by the Institutional Review Board of Peking University Third Hospital. ALL human samples were obtained with signed informed consent by patients voluntarily. We confirmed that all experimental procedures in this study were accordance with the relevant guidelines and regulations. All chemicals were purchased from Sigma Aldrich Co. (Shanghai, China), unless otherwise indicated.

### Collection of human oocytes, embryos and human tissues

In the present study, we obtained GV oocytes from couples undergoing ICSI cycles because of male factor OR male factor–induced infertility. Before ICSI manipulation, all of the oocytes from these couples were digested by hyaluronidase (80 IU/ml) treatment. If GV oocytes were present, the embryologist would inform the couples, who independently decided whether to donate these immature oocytes to the present study or allow them to be destroyed. When the couples decided to donate them to the present study, they would sign the informed consent voluntarily. In total, 260 GV-stage oocytes were used in the present study. On day 3 of the ICSI cycle, human embryos were collected by the embryologist, and those that were considered to be of poor quality and not in accord with the criteria set for embryo transfer were donated with the informed consent from the patients. After legal voluntary termination of pregnancy because of maternal physical disorders, tissues from three female fetuses were collected with informed consent at 19–22 weeks gestation. Adult human ovaries were collected from a patient whose bilateral ovaries were excised because of ovarian cancer. All of these samples were collected at Peking University Third Hospital.

### RNA isolation, cDNA synthesis and qPCR analysis

Total RNA was extracted from the tissues of three fetuses using TRIzol reagent (15596018, Invitrogen, USA) according to the manufacturer’s instructions. cDNA was synthesized using the First Strand cDNA Synthesis kit (Thermo Fisher, Marietta, OH, USA), according to the manufacturer’s instructions.

cDNA from single oocytes or embryos was prepared using a commercial kit (Cat. No. 634854, Clontech Laboratories, USA). A single oocyte or embryo was lysed in lysis buffer, and then the first-strand cDNA was immediately synthesized and tailed without extracting total RNA. Then full-length double-strand cDNA was amplified by long-distance PCR and purified with a QIAquick^®^ PCR Purification kit (Cat. No. 28106, QIAGEN, German). cDNA from tissue samples or single oocytes (or embryos) was used for qPCR using an ABI 7500 machine (Applied Biosystems, USA). The relative expression of *ECAT1* was normalized to expression of *GAPDH*. [Primer sequences are shown in Supplementary Table S1.

### Immunohistochemistry

Fetal and adult ovaries were fixed in 10% formalin. The fixed ovaries were embedded in paraffin, and 6-μm-thick sections of the tissue were prepared. The sections were deparaffinized in xylene and then rehydrated in a graded ethanol series. To expose antigens, the sections were incubated with citrate buffer (pH 6.0) for 20 min at 98 °C and cooled to room temperature, and then 0.3% H_2_O_2_ was used to block endogenous peroxidase activity with a 10-min incubation. The sections were then incubated with rabbit polyclonal anti-ECAT1 (1:400; ab126339, Abcam, UK) overnight at 4 °C. After being washed three times (2 min per wash) in PBS, the sections were incubated for 20 min at room temperature with horseradish peroxidase–conjugated secondary antibodies (PV-9001, ZSGB Biotechnology, China). After three washes in PBS, DAB staining and hematoxylin counter-staining were performed. Finally, an ethanol series and xylene were used to dehydrate the sections. For negative controls, sections were incubated in PBS instead of primary antibody. Slides were photographed using a Nikon ECLIPSE 80i microscope with a Nikon DS-Ri1 camera (Nikon Corporation, Japan).

### Immunofluorescence and signal intensity analysis

After collection, the oocytes and embryos were fixed in 4% paraformaldehyde in DPBS for 30 minutes. The oocytes and embryos were then washed in washing buffer three times (5 min per wash) and were permeabilized with 0.5% Triton X-100 for 30 minutes, followed by three washes (5 min per wash) in washing buffer. Next, the oocytes and embryos were blocked with 1% BSA for 30 minutes at room temperature and incubated with anti-ECAT1 (1:200) or FITC-conjugated anti-α-Tubulin (1:100; F2168, Sigma, USA) overnight at 4 °C, followed by three washes (5 min per wash) The oocytes and embryos incubated with anti-ECAT1 were then incubated for 1 h at room temperature with Alexa Fluor 555–conjugated donkey anti–rabbit IgG (1:200; A31572, Molecular Probes, USA) diluted in 1% BSA, followed by three 5-minute washes in washing buffer. Then the oocytes and embryos were incubated with Hoechst 33342 (1:100; H3570, Invitrogen, USA) for 15 minutes, followed by three 5-minute washes in PBS. Finally, the oocytes and embryos were photographed with a 710 LSM laser scanning confocal microscope (Zeiss, German).

To investigate the intensity of immunofluorescence images, four different images from GV oocytes were analyzed using the profile intensity function of the Zen confocal software (Zeiss). Measurements were performed on the five regions indicated in Fig. 2C: a and e were defined as the cortical cytoplasm region, and b–d were defined as the inner cytoplasm region (a/b/c/d/e = 1:1.5:3:1.5:1). After defining each region, all of the data points in each region were averaged for each image, and then averages for each region for each of the four images and a s.e.m. for each region were generated using Microsoft Excel 2010.

### Preparation and microinjection of siRNAs and *in vitro* maturation of oocytes

Gene function in oocytes was detected by siRNA injection methods. Three siRNA powders were diluted in DEPC-treated water and mixed and then injected into the cytoplasm of human GV oocytes. Successful inhibition was confirmed by single-cell qPCR and dot-blot analysis (see below). Oocytes injected with the NC siRNA were used as controls. After microinjection, immature oocytes were cultured in *in-vitro* maturation medium (F231B, Origio, USA). ECAT1 siRNA sequences are shown in Supplementary Table S1.

### Oocyte dot-blot analysis

Eight oocytes from each group were pooled and lysed in 4 μl RIPA lysis buffer (CW2333S, CWBIO, China), and then 2 μl of each lysate was used to generate each dot on a PVDF membrane (EMD Millipore Corporation, USA). The membrane was air dried and then blocked for 1 h in TBST (0.2 M NaCl, 0.1% Tween-20 and 10 mM Tris [pH 7.4]) containing 5% nonfat dry milk. The blocked membranes were incubated with rabbit polyclonal anti-ECAT1 (1:200) in TBST overnight at 4 °C. After incubation, the membranes were incubated with horseradish peroxidase–conjugated anti–rabbit IgG (1:1000; sc-2030, Santa Cruz Biotechnology, USA) in TBST for 1 h at room temperature. After each step, the membrane was washed three times for 5 min each with TBST, and then bound antibody was detected using an enhanced chemiluminescence detection system (Carestream 4000 MM, USA) according to the manufacturer’s instructions.

### Oocyte fertilization and embryo culture

After 24 h *in vitro* maturation, oocytes were fertilized by the ICSI method^48^. The fertilized oocytes were transferred to GM culture medium (LGGG-050, LifeGlobal Group, Canada) and were incubated under 5% CO_2_ and 95% humidity. Successful fertilization was recorded by observing two distinct pronuclei in zygotes. Embryonic development was observed every 12 h, and embryo morphology was recorded every day until blastocyst formation.

### Statistical analysis

The results were analyzed and compared using SPSS 17.0 software (Chicago, IL, USA). For the data on oocyte maturation, embryo development, embryo grading and the number of embryos with chromosome aneuploidy, a χ^2^ method was applied. For the data on the intensity of immunofluorescence and from qPCR, an independent-sample t-test was performed when comparing the data from two groups. P-values < 0.05 were considered to be significantly different. All experiments were repeated at least three times.

## Additional Information

**How to cite this article**: Liu, C. *et al*. ECAT1 is essential for human oocyte maturation and pre-implantation development of the resulting embryos. *Sci. Rep.* **6**, 38192; doi: 10.1038/srep38192 (2016).

**Publisher's note:** Springer Nature remains neutral with regard to jurisdictional claims in published maps and institutional affiliations.

## Supplementary Material

Supplementary Information

## Figures and Tables

**Table 1:** Effects of ECAT1 siRNA injection on maturation rate of human GV oocytes.

Groups	No. of replicates	No. of injected oocytes	No. of matured oocytes (%)
NC siRNAi	8	60	47 (78%)
ECAT1 siRNAi	15	154	83 (54%)*

^*^P < 0.01, chi-square test, compared with the NC group.

**Table 2:** Effects of ECAT1 siRNA injection in human GV oocytes on subsequent oocytes fertilization and embryos cleavage.

Groups	No. of replicates	No. of ICSI oocytes	No. of fertilized oocytes (%)	No. of cleavaged embryos (%)
NC siRNA	5	31	26 (84)	26 (84)
ECAT1 siRNA	4	26	14 (54)*	8 (31)^#^

^* & #^: P < 0.05, chi-square test, compared with the NC group.
