# Supplementary material for: ECAT1 is essential for human oocyte maturation and pre-implantation development of the resulting embryos
Source: Sci Rep. 2016 Dec 5;6:38192. doi: 10.1038/srep38192 (PMC5137016; doi:10.1038/srep38192)
Supplement: Supplementary Information [file srep38192-s1.pdf]

ECAT1 is essential for human oocyte maturation and pre-implantation development of the resulting embryos

Changyu Liu[1], Min Li[2], Tianjie Li[2], Hongcui Zhao[2], Jin Huang[2], Yun Wang[2], Qian Gao[2], Yang Yu[2]* & Qinghua Shi[1]

Running title: ECAT1is key to human oocyte maturation

[1]Molecular and Cell Genetics Laboratory; The CAS Key Laboratory of Innate Immunity and Chronic Disease; Hefei National Laboratory for Physical Sciences at Microscale; School of Life Sciences, University of Science and Technology of China, Hefei 230027, China.

[2]Beijing Key Laboratory of Reproductive Endocrinology and Assisted Reproductive Technology and Key Laboratory of Assisted Reproduction, Ministry of Education, Center of Reproductive Medicine, Department of Obstetrics and Gynecology, Peking University Third Hospital, Beijing 100191, China.

* Corresponding authors

Yang Yu, Ph.D.

Mailing address: Department of Obstetrics and Gynecology, Peking University Third Hospital, No. 49 HuaYuan North Road, HaiDian District, Beijing 100191, the People's Republic of China

Tel:/Fax: +86-10- 82266753

E-mail: yuyang5012@hotmail.com

Table S1 DNA or RNA sequence of qRT-PCR and ECAT1 siRNAs

Sequence Name	Sequence (5’-3’)	Application
ECAT1 Forward	TGATCATGAACCTGGCTGAC	qRT-PCR
ECAT1 Reverse	GCCTCCCGGACTTCTATTG	qRT-PCR
GAPDH Forward	CTTTGGTATCGTGGAAGGACTC	qRT-PCR
GAPDH Reverse	AGTAGAGGCAGGGATGATGT	qRT-PCR
ECAT1 siRNA Sense-1	CAGGAGGACACAAUCAAGATT	RNAi
ECAT1 siRNA Antisense-1	UCUUGAUUGUGUCCUCCUGTT	RNAi
ECAT1 siRNA Sense-2	CCAGCGGUCUUCAAUAGAATT	RNAi
ECAT1 siRNA Antisense-2	UUCUAUUGAAGACCGCUGGTT	RNAi
ECAT1 siRNA Sense-3	GGACCCAGUUACUAGAUUATT	RNAi
ECAT1 siRNA Antisense-3	UAAUCUAGUAACUGGGUCCTT	RNAi
